# *Nocardia transvalensis *keratitis: an emerging pathology among travelers returning from Asia

**DOI:** 10.1186/1471-2334-11-296

**Published:** 2011-10-31

**Authors:** Elodie Trichet, Stéphan Cohen-Bacrie, John Conrath, Michel Drancourt, Louis Hoffart

**Affiliations:** 1 Service d'ophtalmologie, Hôpital de la Timone, Assistance Publique-Hôpitaux de Marseille, Marseille, France; 2 Pôle des Maladies Infectieuses, Assistance Publique-Hôpitaux de Marseille; Unité de Recherche sur les Maladies Infectieuses et Tropicales Emergentes, UMR CNRS 6236, IRD 3R 198, Université de la Méditerranée, IFR 48, Faculté de Médecine, Marseille, France

**Keywords:** Nocardia keratitis, amikacin, infectious keratitis

## Abstract

**Background:**

The incidence rate of *Nocardia *keratitis is increasing, with new species identified thanks to molecular methods. We herein report a case of *Nocardia transvalensis *keratitis, illustrating this emerging pathology among travellers returning from Asia.

**Case presentation:**

A 23-year-old man presented with a 10-week history of ocular pain, redness, and blurred vision in his right eye following a projectile foreign body impacting the cornea while motor biking in Thaïland. At presentation, a central epithelial defect with a central whitish stromal infiltrate associated with pinhead satellite infiltrates was observed. Identification with 16S rRNA PCR sequencing and microbiological culture of corneal scraping and revealed *N. transvalensis *as the causative organism. Treatment was initiated with intensive topical amikacin, oral ketoconazole and oral doxycycline. After a four-week treatment period, the corneal infiltrate decreased so that only a faint subepithelial opacity remained.

**Conclusion:**

*Nocardia *organisms should be suspected as the causative agent of any case of keratitis in travelers returning from Asia. With appropriate therapy, *Nocardia *keratitis resolves, resulting in good visual outcome.

## Background

*Nocardia *spp. keratitis is an aggressive ocular infection, typically following a corneal trauma. The diagnosis is often delayed, which can lead to a corneal scar [1]. While the most commonly identified agents have been *Nocardia asteroides *and *Nocardia brasiliensis *in the pre-molecular area [2], new species are now identified thanks to molecular methods and later two species are now rarely identified as clinical isolation. Herein, we report one case of *Nocardia transvalensis *keratitis, illustrating this emerging pathology among travelers returning from Asia.

### Case presentation

**Figure 1:**
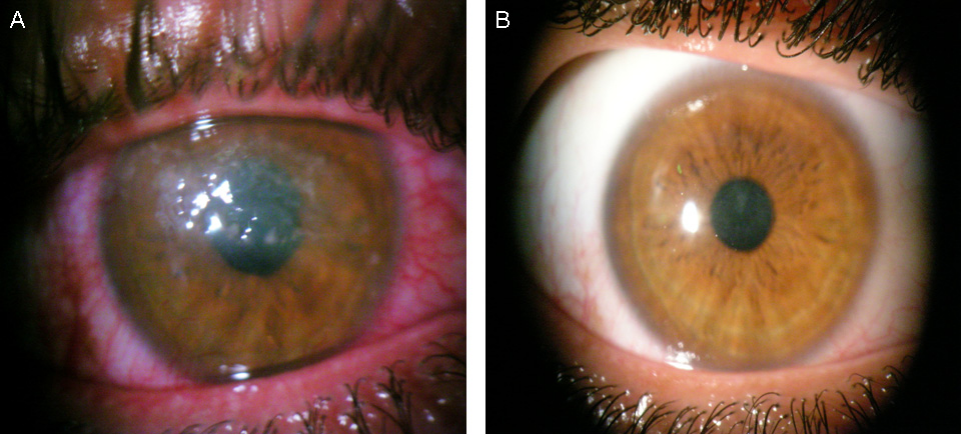
***Nocardia transvalensis *keratitis in a traveler returning from Thailand**. (A) Initial clinical examination showed a whitish infiltrate in the central cornea; (B) Final aspect after 4 weeks of topical amikacin.

A 23-year-old man presented with redness, pain and decreased visual acuity in his right eye following an injury caused by dust while driving a motorcycle in Thailand. Despite topical treatment with prednisolone and neomycin prescribed by a local ophthalmologist, the ocular condition did not improve after four weeks. Ten weeks later, the patient was referred to our ophthalmology department for further management of a persistent corneal ulcer. Upon initial examination, visual acuity was limited to "counting fingers" in the right eye and was 20/20 in the left eye. Slit-lamp examination of the right eye showed a well-defined, dense, whitish infiltrate in the central cornea with an epithelial defect of the same size (Figure 1A). Discrete patches of keratitis with pinhead satellite stromal infiltrates in the margin were also observed, but there was no anterior chamber reaction. Multiple scrapings of the ulcer bed and margins were obtained after topical anesthesia. *N. transvalensis *was cultivated by inoculating 5%-sheep blood agar with the corneal scraping. The culture was incubated at 37°C in a 5% CO_2 _atmosphere. The definite identification was based on partial sequencing of the 16S rRNA gene (over a length of 1.439 nucleotides), which showed a 99.79% sequence similarity with the *N. transvalensis stricto sensu *reference sequence (GenBank accession number GQ217496.1) and a lower 98.96% relatedness to *Nocardia blacklockiae *sequence (GenBank accession number GQ376162.1) and 98.55% to *Nocardia wallacei *sequence (GenBank accession number GQ853074.1). Additional microbiological analyses, including fungal culture and the molecular detection of amoebas and herpes simplex virus (based on the 18S rDNA and DNA polymerase genes respectively), were negative. The patient was given topical amikacin (50 mg/ml) to be applied at hourly intervals in conjunction with oral ketoconazole (200 mg a day) and oral doxycycline (100 mg a day). After a four-week treatment period, the corneal infiltrate decreased so that only a faint subepithelial opacity remained (Figure 1B). Topical corticosteroids were then administered, and the patient recovered a visual acuity of 20/80 three months after the beginning of the treatment. There was no evidence of recurrence during the one-year follow-up period.

In the case reported herein, temporal evidence links the infection and the airborne dust that the patient suddenly felt in his eye while motorbiking in Thailand. Moreover, the patient was not a contact-lens wearer, and he did not have any history of ocular problems. We thus concluded that the patient acquired the infection in Thailand. In this patient, *N. transvalensis *infection was firmly documented by culture and subsequent sequence-based identification. This second reported case of *N. transvalensis *ocular infection [3] indicates that *N. transvalensis *must be added to the list of *Nocardia *species associated with infectious keratitis [2,4]. Of a total of 73 reported cases of *Nocardia *spp. keratitis over the last five years, 67 (92%) have been clearly acquired in individuals with direct links to Asia [2,3,5-16]. Whereas *Nocardia *spp. keratitis is a well-described clinical entity in Asia [1], it is seldom diagnosed in countries outside Asia. A recent visit to Asia therefore provides a clue for clinical diagnosis while waiting for PCR-based confirmation. Laboratory techniques have to be used to analyze every case of infectious keratitis because simple microscopic examination may mistakenly identify the case as fungal keratitis when the histopathology reveals acute-branching septate hyphae similar to those found in fungi. The most frequently noted predisposing factor for *Nocardia *keratitis is trauma, with surgery being the second most common factor. A few cases of *Nocardia *keratitis have also been reported in contact lens wearers, after refractive surgery and after implantation of intracorneal ring segments [1,17].

Topical amikacin is commonly recommended to treat *Nocardia *keratitis [1] based on its *in vitro *antibacterial activity against *Nocardia *organisms [18], its demonstrated corneal penetration and its safety profile [19]. Several authors previously reported that the species *N. transvalensis *in fact comprises of an heterogeneous spectrum of organisms including both amikacin-susceptible and amikacin-resistant organisms, a hallmark of the *N. transvalensis *complex also incorporating the two newly described reported *N. blacklockiae *and *N. wallacei *[20]. In the patient herein reported, intensive tropical application resulted in complete resolution of the infection. One previously published case of *N. transvalensis *keratitis showed a decreased sensitivity to amikacin [3]. In fact, amikacin susceptibility and resistance have been determined on the basis of amikacin concentration achievable in serum during systemic *Nocardia *infections; as for *Nocardia *keratitis, amikacin is used as a topical antibiotic, achieving local concentrations far higher than those achievable during parenteral administration. While amikacin susceptibility profile could be used for the identification of *Nocardia *isolates, it is not useful for the topical treatment of *Nocardia *keratitis.

## Conclusions

The rate of travel of Europeans to tropical regions in Asia for vacation or business has increased dramatically; more than 8 million travelers flew back from Asia to France in 2009. *Nocardia *organisms should be suspected as the causative agent of any case of keratitis in travelers returning from Asia, especially those travelers who experienced a soil-borne corneal trauma and those who are contact lens wearers. Molecular tools may help in making a rapid diagnosis.

## Consent

Written informed consent was obtained from the patient for publication of this report.

## Competing interests

The authors declare that they have no competing interests.

## Authors' contributions

TE wrote the case report; CBS did the laboratory work and wrote the manuscript; CJ took care of the patient; DM interpreted the data and wrote the manuscript; HL took care of the patient and wrote the manuscript. All authors read and approved the final version of the manuscript.

## Pre-publication history

The pre-publication history for this paper can be accessed here:

http://www.biomedcentral.com/1471-2334/11/296/prepub
